# Management of post-traumatic femoral defects with a monorail external fixator over an intramedullary nail

**DOI:** 10.1007/s00590-021-03082-1

**Published:** 2021-08-06

**Authors:** Victor Lu, James Zhang, Andrew Zhou, Matija Krkovic

**Affiliations:** 1 School of Clinical Medicine, University of Cambridge, Cambridge, CB2 0SP UK; 2 Department of Trauma and Orthopaedics, Addenbrooke’s Hospital, Cambridge, CB2 0QQ UK

**Keywords:** Monorail, Bone transport, Femur, Bone defect, Trauma

## Abstract

**Purpose:**

The management of limb-length discrepancy secondary to traumatic femoral bone loss poses a unique challenge for surgeons. The Ilizarov technique is popular, but is associated with long external fixator time and many complications. This retrospective study assessed outcomes of post-traumatic femoral defects managed by monorail external fixation over an intramedullary nail.

**Methods:**

Eight patients were included from October 2015 to May 2019 with post-traumatic femoral defects that underwent treatment with monorail fixator-assisted intramedullary nailing. Primary outcome was time to bone union and bone results according to ASAMI classification. Secondary outcomes were lengthening index, consolidation time and index, external fixator index (EFI), time to partial weight bearing(PWB) and full weight bearing (FWB), and complications. Patient reported outcome measures including EQ-5D-5L, SF-36, Oxford knee scores (OKS), and Oxford hip scores (OHS) were recorded after recovery.

**Results:**

Mean follow-up time was 227 weeks. Average bone defect size was 9.69 cm. Average consolidation time and index were 11.35 months and 1.24 months/cm, respectively. Mean lengthening and external fixator index were 20.2 days/cm and 23.88 days/cm, respectively. On average, patients achieved FWB and bone union 56.25 weeks and 68.83 weeks after bone transport initiation, respectively. Two patients had docking site non-union, five patients had pin site infections, and two patients had osteomyelitis. EQ-5D-5L and EQ-VAS scores were compared to UK population norms (p = 0.104, p = 0.238, respectively). Average OKS was 32.17 and OHS was 34.00.

**Conclusion:**

Monorail external fixation over an intramedullary nail is an effective option for post-traumatic femoral defects, reducing external fixator time and returning patients’ quality of life to a level comparable with the normal population.

## Introduction

The management of limb-length discrepancy secondary to traumatic bone loss poses a unique challenge for surgeons. The first mention of external fixators for bone transport was by Ilizarov, utilising distraction osteogenesis [1]. It is a popular method for managing complex femoral non-union; however, it can lead to docking site non-union, pin site infections, knee stiffness, and has low patient tolerance particularly for upper limb and thigh injuries [2].

Other techniques using external fixators have been described, such as lengthening with external fixator followed by plating, which prevents bending of newly formed bone after frame removal. Despite lowering external fixator duration, this increased the incidence of varus deformities [3]. Lengthening and then nailing (LATN) also decreased external fixator time and protected against re-fracture [1]; these outcomes were supported in a subsequent meta-analysis [4].

This study presents our protocol and outcomes for managing femoral diaphyseal segmental bone defects by monorail external fixation over an intramedullary nail.

## Methodology

The Trauma and Orthopaedics database was retrospectively reviewed for comminuted femoral fractures with bone defects treated with monorail external fixator. Eight patients were included between January 2013 and January 2021. Six were involved in road traffic accidents, and two suffered gunshot wounds. All were male and suffered post-traumatic bone defects (PBDs), specifically severe fractures with acute bone loss (grades 3A or 3B on the Gustilo–Anderson classification), which were temporarily stabilised with open reduction internal fixation (ORIF), prior to bone transport with the monorail external fixator.

Primary outcome was time to bone union, and bone results according to Association for the Study and Application of the Method of Ilizarov (ASAMI) classification. The excellent bone result was union, no infection, deformity of less than 7°, limb-length discrepancy < 2.5 cm. The good bone result was union with two out of three aforementioned criteria. The fair result was union with one of the three. The poor result was non-union or re-fracture or none of the three. Secondary outcomes were lengthening index, consolidation time and index, external fixator index (EFI), time to partial weight bearing (PWB) and full weight bearing (FWB), and complications.

*Lengthening index* was defined as total duration of bone transport per centimetre gain in limb length. *Consolidation time* was defined as time from corticotomy to appearance of consolidation in at least three cortices on lateral and anteroposterior radiographs. *Consolidation index* was defined as consolidation time divided by total lengthening. *EFI* was defined as total duration that external fixator was used divided by total lengthening. Upon recovery, patients completed the patient-reported outcome measures (PROM) questionnaires, including EQ-5D-5L, SF-36, Oxford knee score (OKS), and Oxford hip score (OHS) surveys. Scores were compared with the national average, given the lack of preoperative PROM data.

Extracted quantitative data were analysed with IBM SPSS Statistics version 27. Statistical analyses focused on descriptive statistics such as mean, median, and range. One-tailed, one-sample t-tests were performed between PROMs and national average.

This study was retrospectively registered with the clinical audit department on 13 April 2021 with the registration number PRN9742.

### Surgical protocol

All patients had severe fractures with acute bone loss that was temporarily stabilised with ORIF prior to bone transport. Limb-length discrepancy was calculated using contralateral femur as a reference. Existing metalwork was removed and microbiology samples were taken. Prophylactic intravenous gentamicin and flucloxacillin were given; this was stopped if microbiology samples came back negative, or modified based on the culture results. Sclerosed fracture margins were resected until the Paprika sign was seen, which is punctate cortical or cancellous bleeding [5].

DePuy Synthes lateral femoral nails or retrograde/antegrade femoral nails were inserted in a retrograde (4 patients) and antegrade (4 patients) fashion to the Blumensaat’s line. The bone canal was reamed to 2 mm above the nail diameter. The nail was proximally locked in either standard or recon mode, depending on the surgeon’s preference. Bone transport was performed antegrade in two patients and retrograde in six patients; the former required a subtrochanteric corticotomy, whilst the latter required a distal femoral metaphyseal corticotomy. Corticotomy site was pre-drilled to facilitate subsequent osteotomy with intramedullary nail in situ.

**Fig. 1:**
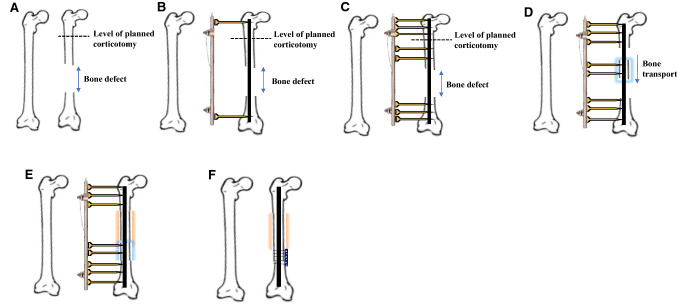
The technique of **distraction osteogenesis** using a monorail external fixator. **A**—Normal femur on the left and a femur with a post-traumatic bone defect on the right. **B**—One pin was inserted into the proximal femur and another pin into the distal femur. A monorail external fixator was assembled to the two pins via connectors. **C**—According to the monorail pin insertion jig, two further pins were then inserted into the proximal and distal fragments, immediately followed by two pins into the transported fragment. **D**—Corticotomy performed. Bone transport (blue halo) begins according to a predetermined lengthening regime. **E**—Bone transport is complete, docking site union is secured, and regenerate is forming (orange halo). **F**—Monorail external fixator removed, and transported fragment is secured in the docking site using a small fragment locking plate.

Fixator pins were inserted under X-ray guidance and were placed parallel to each other, at ~ 30° to the horizontal, passing posterior to the nail. One pin was initially inserted into the proximal fragment, followed by one pin in the distal fragment. A lengthening monorail external fixator was assembled to the two pins via connectors. According to the monorail pin insertion jig, two further pins were then inserted into the proximal and distal fragments, immediately followed by two pins into the transported fragment (Fig. 1). Hydroxyapatite-coated pins were used to lower the chance of pin fixation loosening.

If possible, care was taken to not breach the knee joint capsule. When positioning the pins and connectors, care was taken to allow enough space between connectors, to prevent one connector from colliding with another during bone transport and docking, allowing the transported fragment to dock smoothly.

Following monorail ex-fix assembly, osteotomy was completed using an osteotome at the pre-drilled corticotomy site. Distraction of the osteotomy site between 5 and 10 mm was confirmed on X-ray, and the osteotomy site was then compressed to produce preloaded continuous contact, reducing interfragmentary strain. At the 8th postoperative day, bone transport was started at 0.25 mm four times a day. Patients were followed up every two weeks to radiographically assess callus quality. When lengthening became difficult, the external fixator was readjusted under general anaesthesia to allow smoother passing of transported fragment down the nail. If regenerate was weak, speed was slowed down three weeks later. Regenerate was expected to be weak on anterolateral side of femur, but of reasonable quality on posteromedial side.

When the transported fragment has docked with the distal fragment, the limb was still shorter than the contralateral one. Hence, the nail was kept unlocked at one end, such that further limb lengthening could be done to achieve equal limb length. Only then was the docking site compressed with the monorail, and the nail locked. The transported fragment was then secured in the docking site either utilising nail locking screw options, or with a DePuy Synthes small fragment locking plate. Microbiology samples were taken again. After docking site union was secured, the monorail external fixator was removed.

Standard postoperative care included analgesia, active and passive joint mobilisation exercises, physiotherapy for knee and hip joints, standard pin sites care involving daily sterile gauze and iodine dressing. PWB was allowed during bone transport as per patient’s comfort. FWB started when X-rays confirmed strong regenerate in two dimensions, unless pain was present.

## Results

Average follow-up time was 227 weeks (range 154–331). No patients were lost to follow up. On average, patients had a 10 cm bone defect size (range: 2–16 cm). Average time from emergency department (ED) admission to monorail bone transport procedure was 62 days (13–122 days). Average lengthening index, consolidation time, consolidation index, and EFI were 20 days/cm (range 10–40), 11 months (range 3.58–17.20), 1 months/cm (range 0.89–1.79), and 24 days/cm (range: 11.2–42.0), respectively. All achieved PWB and FWB at an average of 35 weeks (range 15–60) and 56 weeks (range 21–97), respectively.

ASAMI bone scores were excellent in four patients, good in two patients, poor in two patients due to docking site non-union, necessitating reamed intramedullary exchange nailing and docking site compression. Two patients had osteomyelitis caused by *Staphylococcus epidermidis* and *Staphylococcus capitis*. This was treated by six weeks of intravenous vancomycin and teicoplanin in one patient, removal of infected metalwork with antibiotic-loaded calcium sulphate beads in another patient. Five patients had pin site infection, usually affecting proximal medial pin site; all were treated with one week of oral flucloxacillin.

Table 1 summarises patient demographics and Table 2 summarises patient outcomes. Table 3 shows PROMs after recovery. EQ-5D index, EQ-5D VAS, and SF-36 scores were compared against UK population norms and showed no statistically significant differences.

**Table 1:** Demographics

Patient	Age	BMI	Femur laterality	Gustilo–Anderson	Smoking status	Bone defect size (cm)	No. of other injuries	Total surgeries
1	27	24.20	Left	3a	Never	2	2	5
2	23	41.30	Right	3a	Smoker	10	4	4
3	63	30.71	Right	3b	Never	10	0	5
4	58	26.45	Left	3a	Ex-smoker	6	2	3
5	32	30.90	Left	3a	Smoker	4	4	4
6	27	21.35	Right	3a	Smoker	16	2	3
7	29	25.66	Right	3b	Ex-smoker	14.5	2	6
8	43	27.80	Right	3b	Ex-smoker	15	1	6
Average	37.75	28.55	-	-	-	9.69	2.13	4.5

**Table 2:** Patient outcomes

	**Patient 1**	**Patient 2**	**Patient 3**	**Patient 4**	Patient 5*	**Patient 6**	**Patient 7**	Patient 8^*†^	**Mean**
Follow-up time (weeks)	252	217	270	173	331	154	237	178	226.5
Time from ED to monorail ex-fix procedure (weeks)	103	30	13	78	122	57	33	-	62.29
Lengthening index (days/cm)	40	13.3	18.3	10	40	10	20	10	20.2
Consolidation time (months)	3.58	15.60	9.73	5.33	6.25	16.20	17.20	16.90	11.35
Consolidation index (months/cm)	1.79	1.37	0.97	0.89	1.56	1.02	1.19	1.13	1.24
External Fixator index (days/cm)	42	25.9	25	16	39.5	11.2	19.20	12.30	23.88
Time to PWB (weeks)	16	60	54	15	36	36	26	40	35.38
Time to FWB (weeks)	21	97	71	28	60	44	75	54	56.25
Bone union time (weeks)	74	88	100	62	-	36	53	-	68.83
ASAMI bone score results	Excellent	Excellent	Good	Good	Poor	Excellent	Excellent	Poor	-
Complications	- Myositis ossificans—Pin sites bent	- Pin site infection—Docking site non-union	- Cellulitis—Osteomyelitis—Stage 3 osteoarthritis	- Pin site infection—Osteomyelitis	- Docking site non-union—Extreme pain	- Pin site infection—Pins moved position—Plate bending	- Pin site infection	- Pin site infection with cellulitis—Docking site non-union	-

^*^ Patients 5 and 8 still have docking site non-union

^†^ Patient 8 was referred to us for femur lengthening from another hospital

**Table 3:** Patient-reported outcome measures

	**Mean**	**Median**	**SD**	**Range**	**Normal population mean (SD)** [6, 7]	**Study mean compared to UK national mean**	
**Euroqol**							
	Health index (0–1.000)	0.646	0.632	0.124	0.516–0.785	0.91	p = 0.104
	VAS Score (0–100)	67.5	67.5	10.37	50–80	82.5 (17)	p = 0.238
**SF-36**							
	Physical functioning	60.0	57.5	24.9	20–95	92.5 (13.4)	p = 0.281
	Role limitation: Physical	20.8	0	40.05	0–100	91.4 (23.2)	p = 0.164
	bodily pain	61.3	55.0	21.7	45–100	86.3 (17.9)	p = 0.333
	General health	64.8	67.0	19.8	35–85	78.8 (15.7)	p = 0.542
	Vitality	58.3	60.0	26.2	20–95	64.0 (18.2)	p = 0.849
	Social functioning	64.6	75.0	37.4	12.5–100	91.3 (15.8)	p = 0.538
	Role limitation: Emotional	77.8	100	34.4	33.3–100	85.6 (29.3)	p = 0.842
	Mental health	81.3	86.0	15.9	60–100	75.4 (16.3)	p = 0.744
**OHS** (0–48)	34.00	34.00	10.37	17–48	-	-	
**OKS** (0–48)	32.17	29.50	10.76	20–47	-	-	
**OKS-APQ** (0–100)	44.75	40.55	31.73	6–91	-	-	

## Discussion

Bone defects are usually seen after high-energy traumatic incidents, resection of musculoskeletal tumours, and osteomyelitis. Pioneered by Ilizarov, distraction osteogenesis has been shunned by musculoskeletal oncologists due to concerns over poor callus formation during radiochemotherapy [8]. Nevertheless compared to other methods for limb lengthening such as the Masquelet technique, which requires long external fixator times [9] and is deemed unreliable in the lower limb [10], bone transport via distraction osteogenesis has increased in popularity, especially using the lengthening over nail (LON) technique, since it allows early weight bearing and rehabilitation and improves precision of docking site alignment [11].

We have presented a case series of patients with severe fractures that have been initially treated with ORIF, prior to bone transport with the monorail external fixator. This was done based on prior evidence that 30.8% of traumatic segmental long bone defects undergoes spontaneous regeneration [12]. The remaining patients, such as the ones included in this case series, will need definitive bone transport treatment.

The literature contains few studies assessing outcomes of bone transport via monorail assembly with long-term follow-up [13–15]. The clinical outcomes and patient experiences remain obscure. Better understanding of PROMs and long-term outcomes data will enhance surgical confidence, increase long-term reliability of this procedure and help to improve surgical technique.

Like our study, Raschke et al. and Ferchaud et al. maintained homogeneity and only included patients with PBDs [9, 16]. It is important to consider bone defect aetiology, which can produce different outcomes. To showcase this, Schep et al. included 3 patients with bone defects post-debridement for infection, and 12 PBD patients [17]. Healing index was longer in PBD group (1.6 months/cm) than infection group (1.4 months/cm).

Early studies often report outcomes of femoral and tibial bone defects together. The lack of distinction may confuse readers. Ferchaud et al. presented four femoral and two tibial bone defects [9]. Average radiographic consolidation time was longer in tibia bone defects (1.98 months/cm) than femoral bone defects (1.21 months/cm). Average EFI was longer in tibia bone defects (0.65 vs 0.53 months/cm), perhaps due to increased risk of soft tissue invagination in tibia bone defects [18], necessitating decreased bone transport rate and longer time spent in external fixator.

### Functional outcome

Time to bone union was a surprisingly under-reported outcome. In our cohort, patients needed an average of 68.83 weeks (36–100) to achieve docking site union. In retrospective studies, it is difficult to say exactly when union occurred, since no patients can have frequent, evenly spaced radiographs. Especially during COVID-19, follow-up was done virtually, making the gap between X-ray scans even more erratic and prolonged.

ASAMI bone score is a standardised way to report functional outcomes, simplifying comparisons between studies. However, some papers used a ‘modified’ ASAMI bone score. The criteria for ‘excellent bone result’ in Wan et al.’s study is a length discrepancy of < 2 cm [19], rather than the usual criteria of length discrepancy < 2.5 cm. This complicates any fair comparison between different studies.

Our patients needed an average of 35.38 and 56.25 weeks to PWB and FWB, respectively. PWB was defined as being able to support 30–50% of patient’s body without discomfort, whilst FWB was defined as being able to support one’s full body weight without discomfort. Both were measured from time of corticotomy. Direct comparison of PWB and FWB between studies is difficult and can be flawed. The definition of PWB and FWB is heterogeneous, and often studies report it from time of injury. Patients may have had other procedures, before monorail fixator was assembled, which would artificially increase time to PWB/FWB. Even if the definition was standardised, the time from which patient can support 50% of body weight or all their body weight without discomfort is subjective.

Furthermore, the regenerate quality, which influences when a patient can weight bear, is affected by bone transport speed. Agrawal et al. allowed 0.25 mm of transport two to four times daily, with a lengthening index of 13.06 days/cm [14]. Our protocol allowed 0.25 mm of transport four times daily; however, four patients needed slower bone transport due to poor regenerate quality, giving an average lengthening index of 20.2 days/cm. Follow-ups and elective surgeries were often delayed during COVID-19, which contributed to delay in FWB in patients 5 and 6.

Consolidation index and EFI are important measures of functional outcome. Our consolidation index of 1.24 months/cm and EFI of 23.9 days/cm agrees with consolidation index of 41.2 days/cm (~ 1.35 months/cm) by Raschke et al. [16], and EFI of 0.77 months/cm (~ 23.4 days/cm) by Ferchaud et al. [9]. Yet fair comparison with the literature may be difficult due to vague, heterogeneous definitions, or the lack of a definition [20–22]. Two studies utilised the same definition that we used [13, 14], and only then could a fair comparison be made.

### Complications

The management of limb-length discrepancies results in one of the highest complication rates in orthopaedics. Hood et al. reported a 92% complication rate [23], perhaps due to inadequate soft tissue recovery and poor bone blood supply. The Ilizarov method, although widely practiced, is notorious for its high complication rate, which includes failure of distraction osteogenesis, re-fracture, premature consolidation, deformity of newly formed bone, docking site non-union, infections, knee stiffness [2]. A randomised control trial comparing LON to Ilizarov external fixation found the former had lower mean duration of external fixation (52.2 days versus 180.4 days) and fewer complications [24]. A retrospective study showed LON produced fewer complications than internal lengthening techniques such as intramedullary skeletal kinetic distraction [25].

Complications seen in our study are similar to those in the literature, including docking site non-union, acute osteomyelitis, pin site infection, and heterotopic ossification. Kocaoglu et al. saw other complications such as perioperative fractures, equinus contracture, and nail impingement [22]. Pin site infection is commonly reported, with frequencies ranging from 5 to100% [9]. Our study had a rate of 62.5% (n = 5). It is necessary to accept that pin site infection happens regularly, particularly the half pins on the transported fragment, which led to systemic signs of infection in patient 3.

Deep infections are a concern with the LON technique, due to combined use of external and internal fixators. Our study included two patients with osteomyelitis. Other studies report a relatively low deep infection rate of 15% (n = 3) [11], 2.4% (n = 1) [22], and 7% (n = 2) [19]. Nevertheless, a fair comparison between studies is difficult due to the aforementioned baseline differences such as varying bone defect aetiologies and different injury locations.

### PROMs

PROMs are not commonly reported. The long follow-up time in this study makes it ideal to determine quality-of-life measures post-recovery, and no significant difference in EQ-5D-5L and SF-36 scores was seen when compared to UK population norms. The SF-36 results were reported as eight separate domains, respecting its multidimensional nature. Creating a ‘SF-36 total score’ would have erroneously assumed that the best health-related quality of life is a perfect equilibrium between physical and mental components [26]. Nevertheless, a larger sample size and preoperative PROMs are needed to accurately determine if disease burden is reduced using the monorail technique.

Despite satisfactory PROMs in this study, the recent literature suggests that bone transport with self-lengthening intramedullary nails may achieve higher patient satisfaction, especially with regards to cosmetic result, ease of use, and interference with daily activities [27].

### Limitations

To the best of our knowledge, amongst studies investigating treatment of bone defects using monorail external fixator, our study reported the largest variety of outcomes. Our study maintained homogeneity by only including femoral cases with the same bone defect aetiology and provided a detailed description of a surgical protocol that was consistently applied throughout the span of this study. This was performed by only one surgeon, reducing performance bias. Main limitations include the retrospective design, small sample size, and the lack of preoperative PROMs.

## Conclusions

Monorail external fixation over an intramedullary nail is an effective option for post-traumatic femoral defects, reducing duration in external fixator and returning patients’ quality of life to a level comparable with the normal population. The relatively low complication rate and satisfactory outcomes make it an appealing alternative for the management of post-traumatic femoral bone loss. Nevertheless, the recent literature has suggested that self-lengthening intramedullary nails may increase patient satisfaction and decrease complication rates [28].

Given the low prevalence of post-traumatic femoral defects treated with monorail external fixators, future research could include multi-centre studies for a larger sample size, providing a comprehensive report of functional, radiographic, and patient-reported outcomes, with key terms defined. Homogeneity of femoral defect aetiology and location of pathology should be preserved when selecting patients.

## Declarations

### Availability of data and material

The authors confirm that the data supporting the findings of this study are available within the article [and/or] its supplementary materials.
